# The Prognostic Value of Neutrophil-to-lymphocyte Ratio and Monocyte-to-lymphocyte Ratio in Metastatic Gastric Cancer Treated with Systemic Chemotherapy

**DOI:** 10.7150/jca.39575

**Published:** 2020-04-25

**Authors:** Danyang Zhou, Ying Wu, Ying Zhu, Zhenyu Lin, Dandan Yu, Tao Zhang

**Affiliations:** 1 Cancer Center, Union Hospital, Tongji Medical College, Huazhong University of Science and Technology, Wuhan 430022, China; 2 Department of Medical Oncology, Sun Yat-sen University Cancer Center, State Key Laboratory of Oncology in South China, Collaborative Innovation Center for Cancer Medicine, 651 Dongfeng East Rd, Guangzhou, 510060, China

**Keywords:** neutrophil-to-lymphocyte ratio, monocyte-to-lymphocyte ratio, progression-free survival, metastatic gastric cancer, systemic chemotherapy

## Abstract

**Background:** The prognostic value of neutrophil-to-lymphocyte ratio (NLR) and monocyte-to-lymphocyte ratio (MLR) in metastatic gastric cancer (mGC) treated with systemic chemotherapy is largely unknown, especially second-line chemotherapy. We retrospectively investigated the prognostic value of baseline NLR and MLR in the progression of mGC with systemic chemotherapy.

**Methods:** Patients with mGC diagnosed by pathology from January 2010 to December 2018 were identified. Baseline NLR and MLR were collected before treatment. The time to progression during or after first-line therapy from diagnosis (PFS1), and during or after second-line chemotherapy (PFS2) were primary endpoint. Overall survival (OS) was calculated from diagnosis to the date of death or final follow-up.

**Results:** 537 patients with first-line chemotherapy were included in the retrospective study. The cutoff values of NLR and MLR were 2.610 and 0.285, respectively. Pretreatment NLR and MLR were significantly independent prognostic factors for PFS1 (hazard ratio [HR]=1.597, 95% CI 1.261-2.022, *P*<0.001 and HR=1.574, 95% CI 1.239-1.999, *P*<0.001) and OS (HR=1.448, 95% CI 1.030-2.034, *P*=0.033 and HR=1.622, 95% CI 1.148-2.291, *P*=0.006). For 172 patients treated with second-line chemotherapy, the cutoff value of MLR was 0.355 and MLR maintained a significant association with PFS2 (HR=1.589, 95% CI 1.073-2.354, *P*=0.021) in multivariate analysis.

**Conclusions:** Elevated NLR and MLR were markedly related to the worse PFS1 and OS in mGC performed with first-line chemotherapy. In patients with second-line therapy, MLR was more closely connected to prognosis and was a significantly independent prognostic factor for PFS2.

## Introduction

Gastric cancer (GC) ranks the third for cancer deaths and fifth for cancer incidence worldwide^1^. The death and incidence rates are highest in Asia Pacific^2^, ^3^. In China, a large proportion of the patients are diagnosed at an advanced stage due to a lack of specific symptoms^4^, ^5^. Despite advances in diagnoses and treatments, the prognosis for metastatic gastric cancers (mGC) remains dismal^6^. A doublet or triplet chemotherapy regimen based fluoropyrimidine and platinum agents is the standard of care for mGC patients with a negative expression of human epidermal growth factor receptor-2 (HER-2) receptor^7^, ^8^ according to the current international guidelines^9^. Many of patients receiving first-line therapy may relapse, modest survival benefit is shown in patients receiving irinotecan, taxane and ramucirumab over the best supportive care or active agents for these population^10^-^13^. However, there are still some mGC patients with the standard therapies and the significantly different prognoses in clinical practice.

Inflammation is an essential component of the tumor microenvironment, and the changes in inflammatory cells have an important role in tumorigenesis, disease progression and patients' prognosis^14^, ^15^. Peripheral blood tests represent the mirror of the tumor inflammatory conditions. Neutrophil-to-lymphocyte ratio (NLR) and monocyte-to-lymphocyte ratio (MLR), as the peripheral blood parameters, are the systemic inflammatory response indicators that have been widely demonstrated to predict outcomes of several solid malignancies^16^-^21^, including our previous data^22^. An increase in the NLR reflects a state of lymphocytopenia and neutrophilia that leads to a protumor microenvironment^23^. Elevated MLR, in fact, prompts to cancer progression through the inhibition of immune system.

In gastric cancer, these hematological parameters also have significantly prognostic values on patients' survival. Li Chen et al. investigated the prognosis of systemic immune-inflammation index, such as platelet-to-lymphocyte ratio (PLR)^24^, SII (SII= neutrophil (N)*platelet (P)/lymphocyte (L))^25^ and MLR^26^, in patients with GC treated with neoadjuvant chemotherapy. Hua-Long Zheng et al. sought to characterize the effect of the white blood cell to hemoglobin ratio (WHR) on long-term survival after radical gastrectomy^27^. Another study performed by Xiao-dong Chen evaluated the predictive value of PLR for peritoneal metastasis in patients with GC^28^. However, studies on the prognostic values of the NLR and MLR in mGC treated with systemic chemotherapy, especially second-line therapy are rarely reported. Based on this background, the aim of this study was to investigate the prognostic role of the NLR and MLR for survival in mGC patients with first- and second-line strategy.

## Methods

### Patients population

We retrospectively collected clinical data for mGC patients diagnosed pathologically at our Cancer Center between January 2010 and December 2018. Clinical stage of the disease was determined following the 8^th^ American Joint Committee on Cancer guidelines^29^. Patients meeting any of the following criteria were excluded: (1) patients without complete pretreatment baseline parameters; (2) any malignancies besides GC; (3) hematological diseases; (4) evidences of infection or autoimmune diseases; (5) patients without systemic chemotherapy. For all patients, clinical data (age, gender, histology, HER-2 status and chemotherapy regimens) and hematological examination (complete blood count, tumor biomarkers and biochemical parameters) were collected before treatment. Tumor assessment was performed at baseline, at week 8 after chemotherapy, and every 8-12 weeks thereafter, and clinical response was classified according to response evaluation criteria in solid tumors (RECIST 1.1)^30^. All patients included were followed-up regularly until death or study data cutoff (31 December 2018). This retrospective study was approved by the Ethics Committee of Huazhong University of Science and Technology (HUST) in accordance with the ethical standards prescribed by Helsinki Declaration.

### Statistical analysis

Baseline characteristics and treatment strategies of patients were summarized using descriptive statistics. NLR was calculated as the absolute neutrophil count divided by the absolute number of lymphocyte count (NLR=ANC/ALC) and MLR= AMC/ALC. Analysis of receiver operating characteristic (ROC) curves was performed to identify the cutoff value of variables. Median value severed as the cutoff value if area under the curve (AUC) of ROC was less than 0.50. Progression-free survival 1 (PFS1) was determined from diagnosis to disease progression during or after first-line chemotherapy evaluated by imaging, or death (event), or last follow-up (censored). The progression during or after second-line chemotherapy, or death (event), or last follow-up (censored) were defined as progression-free survival 2 (PFS2). Overall survival (OS) was calculated from diagnosis to death (event) or last follow-up (censored). Survival analyses were calculated by the Kaplan-Meier method. Univariate and multivariate analyses were documented using Cox proportional hazards model, and all the significant characteristics on univariate analysis were carried into multivariate analysis. Differences between categorical variables were determined using the Chi-square test. Statistical analysis was performed using SPSS version 21.0. All *P* values were two-sided, and* P* values < 0.05 were considered significant for all statistical analyses.

## Result

### Patients treated with first-line chemotherapy

#### Clinicopathologic characteristics of patients

A total of 537 mGC patients who received regularly first-line therapy between January 2010 and December 2018 had available clinicopathological data and met the inclusion criteria. Clinical and pathological characteristics of patients were presented in Table 1. The median age at time of diagnosis was 55.0 years (range 25-83 years) and there were 216 (40.2%) women and 321 (59.8%) men. Mean NLR and MLR were 3.060 and 0.320, respectively. The main first-line chemotherapy schedule was the combination of platinum and fluorouracil (80.1%). The median PFS1 and OS were 6.80 and 13.42 months, respectively. The last follow-up time was 31 December 2018.

#### Prognostic role of baseline NLR and MLR for PFS1 and OS

**Figure 1:**
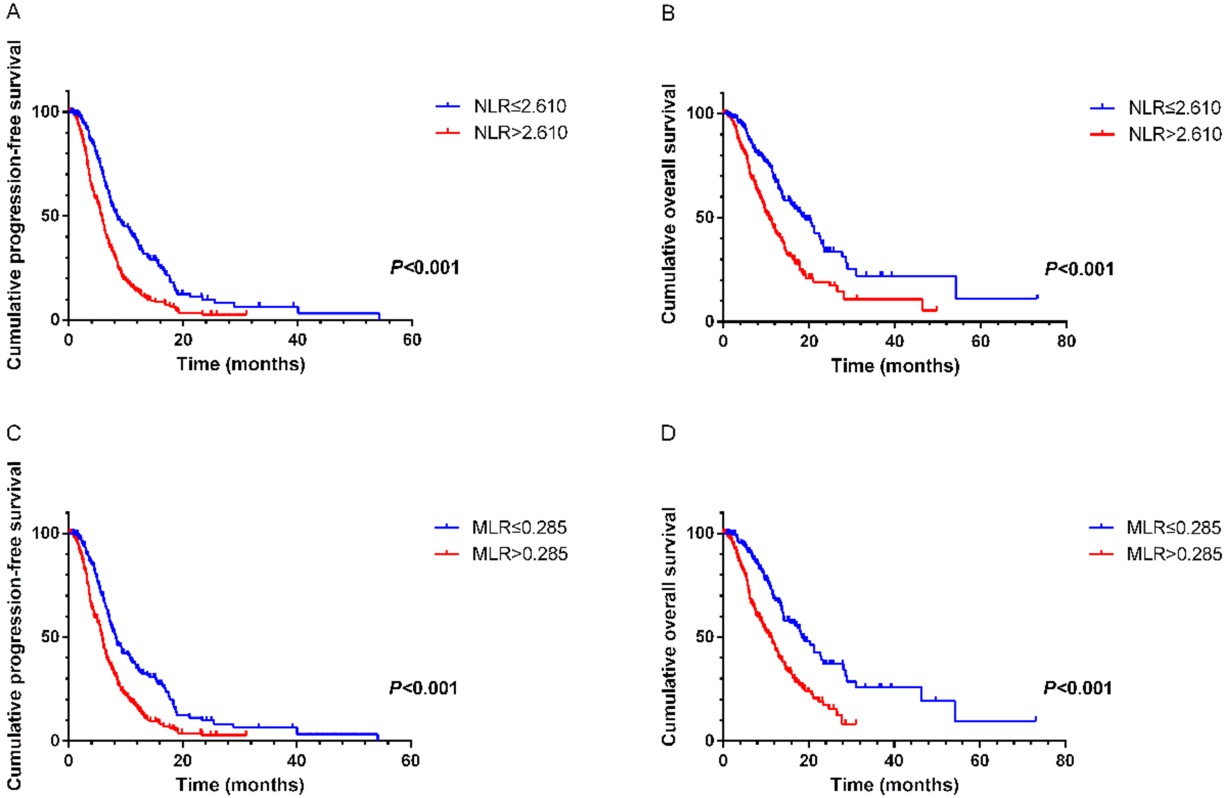
** A.** Kaplan-Meier curve for PFS1 of mGC patients with first-line chemotherapy stratified by NLR. **B.** Kaplan-Meier curve for OS of mGC patients with first-line chemotherapy stratified by NLR. **C.** Kaplan-Meier curve for PFS1 of mGC patients with first-line chemotherapy stratified by MLR. **D.** Kaplan-Meier curve for OS of mGC patients with first-line chemotherapy stratified by MLR.

NLR and MLR were calculated based on the recommended cutoff values of 2.610 and 0.285 from ROC curves, respectively. On univariate analysis the following variables were found to have prognostic value for PFS1: NLR (hazard ratio [HR]=1.897, 95% CI 1.529-2.353, *P*<0.001), MLR (HR=1.795, 95% CI 1.443- 2.233, *P*<0.001), CA125 (HR=1.691, 95% CI 1.354-2.113, *P*<0.001), CA199 (HR=1.401, 95% CI 1.129-1.737, *P*= 0.002), alkaline phosphatase (ALP) (HR=1.268, 95% CI 1.031-1.559, *P*=0.025). On multivariate analysis showed that elevated NLR (HR=1.597, 95% CI 1.261- 2.022, *P*<0.001), MLR (HR=1.574, 95% CI 1.239-1.999, *P*<0.001), CA125 (HR=1.559, 95% CI 1.238-1.964, *P*<0.001) and CA199 (HR=1.256, 95% CI 1.003-1.572, *P*=0.047) were the variables independently associated with shortened survival (Table 2). Similar results were revealed in the relationships of these factors with OS. Multivariate analysis demonstrated that pretreatment NLR (HR=1.448, 95% CI 1.030-2.034, *P*=0.033), MLR (HR=1.622, 95% CI 1.148-2.291, *P*=0.006) and CA125 (HR=1.675, 95% CI 1.267-2.215, *P*<0.001) were significantly correlated with OS (Table 3). Kaplan-Meier survival curves also suggested that NLR and MLR had close links with PFS1 and OS (Figure 1).

#### Associations of NLR and MLR with other clinicopathologic parameters

Baseline NLR and MLR as promising prognostic factors in mGC patients conducted with first-line chemotherapy, we subsequently performed a comparison of the clinicopathologic characteristics according to NLR and MLR, respectively. The following variables related to elevated NLR: high CA125 (*P*<0.001), CEA (*P*<0.001), globulin (GLB) (*P*=0.031) and ALP (*P*=0.001). CA125 (*P*<0.001), albumin (ALB) (*P*=0.011) and ALP (*P*=0.002) had significant relations with MLR.

### Patients treated with second-line chemotherapy

#### Clinicopathologic characteristics of patients

Among 537 mGC patients with first-line chemotherapy, 172 (32.0%) patients conducted the second-line chemotherapy after disease progression which is consistent with previous statistics^31^. Could NLR and/or MLR be beneficial in predicting the development of tumor for these patients? Baseline data of 172 patients were shown in Table 4. The main first-line schedule was also a doublet chemotherapy regimen-based fluorouracil and platinum agents (87.2%). The second-line regimen was following: chemotherapy based taxane (45.3%) and chemotherapy-based platinum (16.9%), chemotherapy-based irinotecan (16.3%) and others (21.5%). The mean values of NLR and MLR were 3.030 and 0.310, respectively. The median month of PFS2 was 3.70 months.

#### Prognostic role of baseline NLR and MLR for PFS2

**Figure 2:**
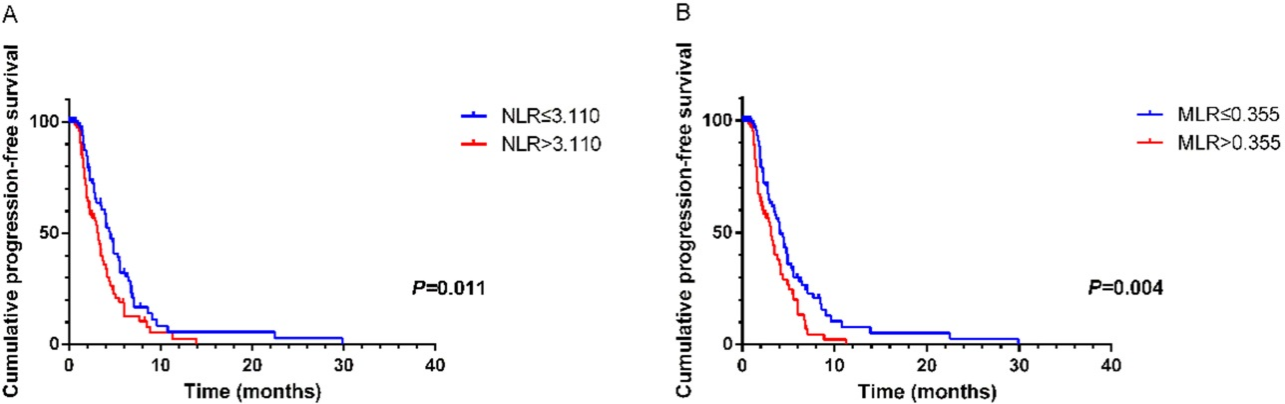
Kaplan-Meier curve for PFS2 of mGC patients with second-line chemotherapy stratified by NLR** (A)** and MLR **(B)**.

According to ROC curves, the cutoff values of NLR and MLR were 3.110 and 0.355, respectively. Significant characteristics on univariate analysis (NLR (HR=1.588, 95% CI 1.102-2.287, *P*=0.013), MLR (HR=1.703, 95% CI 1.178-2.463, *P*=0.005) and CA125 (HR=1.602, 95% CI 1.075-2.389, *P*=0.021)) were carried into multivariate analysis. MLR (HR=1.589, 95% CI 1.073-2.354, *P*=0.021) and CA125 (HR=1.564, 95% CI 1.043-2.346, *P*=0.031)) were significantly associated with PFS2 by multivariate analysis (Table 5). Using Kaplan-Meier analysis, elevated NLR and MLR predicted a shorter disease progression, although NLR was not an independent prognostic factor (Figure 2).

#### Associations of MLR with other clinicopathologic parameters

In analyzing the correlation between MLR and clinicopathologic factors, CA125 (*P*=0.098) seemed to be different among the two groups stratified by MLR, but not statistically significance.

## Discussion

In this study, we aimed to identify clinically useful inflammation-based prognostic indicators for individualized treatments in mGC patients undergoing systemic chemotherapy and most likely to help these patients to benefit from current chemotherapeutic strategies. On the base of analysis on patients with first-line chemotherapy, we further explored the prognostic value of systemic inflammatory response indicators, namely NLR and MLR in patients accepted with second-line chemotherapy. Present study demonstrated that baseline NLR and MLR were independently predictive factors for mGC treated with first-line chemotherapy. For mGC patients performed with second-line therapy, MLR possessed better prognostic value for disease progression and therapeutic efficiency than NLR.

It is widely recognized that inflammation promotes the development of tumor, such as Helicobacter pylori infection and the risk of gastric cancer^32^. On the other hand, tumor can also trigger regional inflammatory responses and release proinflammatory cytokines leading to the formation of an inflammatory microenvironment^33^-^35^. Elevated NLR reflects a state of neutrophilia and/or lymphocytopenia and high MLR presents increased monocyte and/or decreased lymphocyte. Neutrophils and monocytes are linked to induce angiogenesis, inflammation and pro-tumor immune response by secreting a variety of cytokines and cytotoxic mediators^36^-^38^. Tumor-infiltrating lymphocytes, as a type of lymphocytes, are the most important mediators of an anti-tumor immune response^39^, ^40^.

In our study, elevated NLR and MLR predict the worse PFS1 and OS which is constant with the previous researches^20^, ^41^. For mGC patients, the survival analysis of systemic chemotherapy is superior to best supportive care in several randomized studies^42^, ^43^. Therefore, subgroup analysis of prognostic factors for mGC treated with systemic chemotherapy are more clinically valuable than that of advanced patients regardless of treatment or not. For patients with first-line chemotherapy, NLR and MLR serves as prognostic factors to evaluate the treatment effectiveness and the survival. Van Soest RJ et al.^44^, Bruix J et al.^45^ and Choi YH et al.^46^ demonstrated the similar results in solid cancer with systemic chemotherapy. NLR and MLR are also related to CA125^47^ and ALP^48^ that are widely demonstrated to associate with the prognosis of solid cancers. Tumor markers are related to tumor burden^49^ and elevated serum ALP are known to be associated with poor survival in locally advanced and metastatic cancers^50^.

Many of patients receiving first-line therapy relapse over time and second-line treatment remains the only available option for disease progression^51^. For these patients, the prognostic factors for disease progression and therapeutic efficiency are controversial. In present study, MLR is a significantly independent factor in mGC with second-line chemotherapy. Unfortunately, NLR has no significant association with disease progression in these people. However, little is known about the mechanism of NLR and MLR and its association with prognosis in metastatic cancer with chemotherapy. Neutropenia and its complications remain major toxicities related to myelosuppressive chemotherapy in several solid tumors^52^, ^53^. With the increasing cycles of chemotherapy, bone marrow toxicities accumulate and immune status alters^54^, ^55^. As responders in inflammation, neutrophils rapidly migrate between the blood stream and the tissue. Recent discoveries have revealed the ability of these cells to shape the immune response by playing an important role at the interface between innate and adaptive immunity^56^. This may partly explain why NLR lose the prognosis in mGC with second-line therapy. Reported previously, hepatocyte growth factor receptor (c-Met) 57, immune checkpoints (PD-1 and PD-L1)^58^, BRCA mutations^59^ and fibroblast growth factor receptors (FGFR) and their ligands^60^ are the most studied biomarkers for survival and make breakthroughs. MLR, as a saving-time, inexpensive and valid biomarker, has its advantages in clinical practice. To the best of our knowledge, our current study is the first to show that MLR is a promising factor for evaluating the prognosis in patients with mGC who undergone second-line chemotherapy.

Regrettably, NLR and MLR have no significate link to HER-2 expression both first- and second-line chemotherapy. Other prognostic markers need to be explored for mGC with a positive expression of HER- 2 receptor who conducted with systemic treatment.

Nevertheless, we should be clear that there are some limitations in addition to the single institution and retrospective study design. The present findings need to be validated using a larger cohort of patients and subgroup analysis between different second-line regimens.

## Conclusion

Although systemic chemotherapy is the first choice for mGC patients, the prognostic factors for survival and efficiency are yet to be explored. Pretreatment NLR and MLR were markedly related to the progression and survival in mGC with first-line chemotherapy, while MLR are more promising in patients with second-line treatment. NLR and MLR, as simple, inexpensive and readily available biomarkers, if proper combination and application, help predict response to chemotherapy and survival in mGC patients with systemic chemotherapy.

## Figures and Tables

**Table 1:** General characteristics of mGC patients undergoing first-line chemotherapy (n=537)

Variable	Value
Age (Year)	55.0 (25-83)
Gender (Female/male)	216/321
Histology (Well/moderately/poorly differentiated/unknown)	6/38/303/190
HER-2 (Positive) (available in 210 pts)	22 (10.5%)
First-line chemotherapy	
Platinum + fluorouracil	430 (80.1%)
Others	107 (19.9%)
NLR	3.060 (0.12-58.23)
PLR	196.0 (21-886)
MLR	0.320 (0.05-1.91)
CA125 (U/ml)	30.75 (3.0-4853.6)
CA199 (U/ml)	17.70 (1.0-12000.0)
CEA (ug/L)	3.45 (0.5-11112.9)
ALB (g/L)	37.70 (15.9-54.4)
GLB (g/L)	26.70 (15.1-51.7)
ALP (U/L)	73.0 (27-3229)
Progress-free survival 1	6.80 (0.1-54.2)
Overall survival	13.42 (0.1-73.2)

Abbreviations: NLR: neutrophil-to-lymphocyte ratio; PLR: platelet-to-lymphocyte ratio; MLR: monocyte-to-lymphocyte ratio; ALB: albumin; GLB: globulin; ALP: alkaline phosphatase; Progress-free survival 1: the time to progression after first-line from diagnosis.

**Table 2:** Cox proportional hazard regression analysis of progression-free survival in mGC patients undergoing first-line chemotherapy (n=573)

Faction	Univariable			Multivariable	
	HR (95% CI)	*P*		HR (95% CI)	*P*
Age (> 55.0 vs ≤ 55.0)	0.976 (0.795-1.198)	0.818			
Gender (M vs F)	0.997 (0.809-1.228)	0.975			
Histology (Well vs moderately vs poorly differentiated)	1.192 (0.876-1.624)	0.264			
HER-2 (Positive vs negative)	1.330 (0.776-2.279)	0.300			
NLR (> 2.610 vs ≤ 2.610)	1.897 (1.529-2.353)	**< 0.001**		1.597 (1.261-2.022)	**< 0.001**
PLR (> 284.0 vs ≤ 284.0)	1.227 (0.960-1.569)	0.103			
MLR (> 0.285 vs ≤ 0.285)	1.795 (1.443-2.233)	**< 0.001**		1.574 (1.239-1.999)	**< 0.001**
CA125 (> 25.90 vs ≤ 25.90 U/ml)	1.691 (1.354-2.113)	**< 0.001**		1.559 (1.238-1.964)	**< 0.001**
CA199 (> 17.70 vs ≤ 17.70 U/ml)	1.401 (1.129-1.737)	**0.002**		1.256 (1.003-1.572)	**0.047**
CEA (> 4.55 vs ≤ 4.55 ug/L)	1.205 (0.975-1.489)	0.085			
ALB (> 39.25 vs ≤ 39.25 g/L)	0.928 (0.742-1.161)	0.515			
GLB (> 26.70 vs ≤ 26.70 g/L)	1.177 (0.953-1.452)	0.130			
ALP (> 78.5 vs ≤ 78.5 U/L)	1.268 (1.031-1.559)	**0.025**		1.144 (0.911-1.437)	0.246

Abbreviations: NLR: neutrophil-to-lymphocyte ratio; PLR: platelet-to-lymphocyte ratio; MLR: monocyte-to-lymphocyte ratio; ALB: albumin; GLB: globulin; ALP: alkaline phosphatase. *P* less than 0.05 is statistically significant.

**Table 3:** Cox proportional hazard regression analysis of overall survival in mGC patients undergoing first-line chemotherapy (n=573)

Faction	Univariable			Multivariable	
	HR (95% CI)	*P*		HR (95% CI)	*P*
Age (> 55.0 vs ≤ 55.0)	1.157 (0.899-1.488)	0.257			
Gender (M vs F)	0.909 (0.706-1.171)	0.461			
Histology (Well vs moderately vs poorly differentiated)	0.922 (0.609-1.396)	0.701			
HER-2 (Positive vs negative)	1.240 (0.659-2.331)	0.505			
NLR (> 2.610 vs ≤ 2.610)	2.034 (1.555-2.661)	**< 0.001**		1.448 (1.030-2.034)	**0.033**
PLR (> 284.0 vs ≤ 284.0)	1.452 (1.080-1.953)	**0.014**		1.016 (0.733-1.406)	0.926
MLR (> 0.285 vs ≤ 0.285)	2.218 (1.678-2.930)	**< 0.001**		1.622 (1.148-2.291)	**0.006**
CA125 (> 25.90 vs ≤ 25.90 U/ml)	1.909 (1.453-2.508)	**< 0.001**		1.675 (1.267-2.215)	**< 0.001**
CA199 (> 17.70 vs ≤ 17.70 U/ml)	1.275 (0.982-1.655)	0.068			
CEA (> 4.55 vs ≤ 4.55 ug/L)	1.545 (1.195-1.998)	0.001			
ALB (> 39.25 vs ≤ 39.25 g/L)	0.759 (0.574-1.003)	0.052			
GLB (> 26.70 vs ≤ 26.70 g/L)	0.930 (0.715-1.210)	0.591			
ALP (> 78.5 vs ≤ 78.5 U/L)	1.156 (0.896-1.491)	0.265			

Abbreviations: NLR: neutrophil-to-lymphocyte ratio; PLR: platelet-to-lymphocyte ratio; MLR: monocyte-to-lymphocyte ratio; ALB: albumin; GLB: globulin; ALP: alkaline phosphatase. *P* less than 0.05 is statistically significant.

**Table 4:** General characteristics of mGC patients undergoing second-line chemotherapy (n=172)

Variable	Value
Age (Year)	53.0 (27-76)
Gender (Female/male)	72/100
Histology (Well/moderately/poorly differentiated/not known)	2/16/98/56
HER-2 (Positive) (available in 73 pts)	8 (11.0%)
First-line chemotherapy	
Platinum + fluorouracil	150 (87.2%)
Others	22 (12.8%)
Second-line chemotherapy	
Chemotherapy-based taxane	78 (45.3%)
Chemotherapy-based platinum	29 (16.9%)
Chemotherapy-based irinotecan	28 (16.3%)
Others	37 (21.5%)
NLR	3.030 (0.92-12.91)
PLR	196.0 (38-620)
MLR	0.310 (0.08-1.35)
CA125 (U/ml)	34.60 (6.5-1061.7)
CA199 (U/ml)	18.10 (1.0-12000.0)
CEA (ug/L)	2.80 (0.5-11112.9)
ALB (g/L)	37.90 (20.0-54.4)
GLB (g/L)	26.35 (16.5-48.7)
ALP (U/L)	74.5 (37-468)
Progress-free survival 2	3.70 (0.1-29.9)

Abbreviations: NLR: neutrophil-to-lymphocyte ratio; PLR: platelet-to-lymphocyte ratio; MLR: monocyte-to-lymphocyte ratio; ALB: albumin; GLB: globulin; ALP: alkaline phosphatase; Progress-free survival 2: the time to progression after second-line chemotherapy.

**Table 5:** Cox proportional hazard regression analysis of progression-free survival in mGC patients undergoing second-line chemotherapy (n=172)

Faction	Univariable			Multivariable	
	HR (95% CI)	*P*		HR (95% CI)	*P*
Age (> 53.0 vs ≤ 53.0)	0.950 (0.662-1.364)	0.781			
Gender (M vs F)	1.262 (0.874-1.820)	0.214			
Histology (Well vs moderately vs poorly differentiated)	0.896 (0.555-1.449)	0.655			
HER-2 (Positive vs negative)	0.953 (0.375-2.420)	0.919			
NLR (> 3.110 vs ≤ 3.110)	1.588 (1.102-2.287)	**0.013**		1.435 (0.975-2.112)	0.067
PLR (> 129.0 vs ≤ 129.0)	0.973 (0.630-1.503)	0.903			
MLR (> 0.355 vs ≤ 0.355)	1.703 (1.178-2.463)	**0.005**		1.589 (1.073-2.354)	**0.021**
CA125 (> 34.60 vs ≤ 34.60 U/ml)	1.602 (1.075-2.389)	**0.021**		1.564 (1.043-2.346)	**0.031**
CA199 (> 18.10 vs ≤18.10 U/ml)	1.171 (0.799-1.717)	0.418			
CEA (> 2.80 vs ≤ 2.80 ug/L)	1.386 (0.950-2.023)	0.091			
ALB (> 38.00 vs ≤ 38.00 g/L)	0.707 (0.478-1.045)	0.082			
GLB (> 26.35 vs ≤ 26.35 g/L)	1.078 (0.742-1.566)	0.692			
ALP (> 74.5 vs ≤ 74.5 U/L)	1.259 (0.875-1.814)	0.215			

Abbreviations: NLR: neutrophil-to-lymphocyte ratio; PLR: platelet-to-lymphocyte ratio; MLR: monocyte-to-lymphocyte ratio; ALB: albumin; GLB: globulin; ALP: alkaline phosphatase. *P* less than 0.05 is statistically significant.
